# Case report: Autoimmune encephalitis associated with anti-CASPR2 antibody mimicking cerebral infarction

**DOI:** 10.3389/fimmu.2023.1041664

**Published:** 2023-01-26

**Authors:** Ziqi Chen, Jing Tang

**Affiliations:** Department of Radiology, West China Hospital of Sichuan University, Chengdu, China

**Keywords:** autoimmune, contactin-associated protein-like 2, encephalitis, stroke, magnetic resonance imaging

## Abstract

Autoimmune encephalitis associated with antibody against contactin-associated protein-like 2 (CASPR2) varies in its clinical presentation. The disease is difficult to distinguish from some other conditions without testing for anti-CASPR2 antibody in blood serum or cerebrospinal fluid. Cerebral lesions are typically detected by magnetic resonance imaging (MRI) in the medial temporal lobe or hippocampus. Here, we describe a patient with anti-CASPR2 antibody autoimmune encephalitis whose imaging manifestations mimicked infarction in the left frontal lobe. The 48-year-old man reported memory loss, convulsions, and disturbed consciousness one day after drinking wine. The right upper arm showed reduced autonomous movement after painful stimuli, and MRI showed abnormal hyperintensities in the left frontal lobe on T2 and fluid-attenuated inversion recovery sequences, restricted diffusion, and decreased cerebral blood flow, mimicking acute cerebral infarction. Contrast-enhanced T1-weighted MRI showed gyral enhancement involving the cortex and subcortical white matter. Computed tomography angiography did not identify culprit blood vessels. Symptoms did not improve with anti-platelet or lipid-lowering therapy. Screening for serum antibodies associated with autoimmune encephalitis detected antibody against CASPR2, and intravenous immunoglobulin therapy substantially improved symptoms. This case provides the first indication that anti-CASPR2 antibody-associated autoimmune encephalitis can manifest as involvement of the cortex and subcortical white matter in the frontal lobe based on MRI. It emphasizes the need for thorough investigation, including analysis of potential autoimmunity, of patients whose imaging findings mimic ischemic infarction.

## Introduction

Contactin-associated protein-like 2 (CASPR2) is a transmembrane adhesion protein of the neurexin superfamily, and it mediates intercellular interactions. It ensures the proper localization of voltage-gated potassium channels (1, 2) in the node of Ranvier, initial axon segment, and synapses for action potential propagation (3, 4). Autoimmunity against CASPR2 is involved in the pathogenesis of several neurological disorders. The most common is autoimmune encephalitis, which is a limbic encephalitis with symptoms including mental and behavioral disorders, cognitive impairment, memory deterioration, and epilepsy (5, 6). Other neurological disorders include Morvan’s syndrome, acquired peripheral hyperexcitability (Isaacs’ syndrome), cerebellar syndromes and, more rarely, movement disorders (3, 7).

The medial temporal lobe appears to be the region of the central nervous system most vulnerable to anti-CASPR2 antibodies, with patients presenting unilateral or bilateral T2 hyperintensities on magnetic resonance imaging (MRI) (6, 8, 9). Here, we report a patient with autoimmune encephalitis associated with the presence of anti-CASPR2 antibody whose MRI showed frontal lobe lesions mimicking cerebral infarction.

## Case description

A 48-year-old Han Chinese man was admitted to our emergency department because of transient memory loss and depressed mood, followed by two episodes of seizures and loss of consciousness. These symptoms manifested at one day after he drank wine. No fever, headache, or urinary incontinence was reported, but his family reported that he had a history of hypertension, a diagnosis of diabetes more than 10 years prior, and progressively worsening memory over the last four years, for which he did not seek treatment.

On admission, the patient’s vital signs were stable. Neurological examination indicated a lethargic state and inability to follow the clinician’s requests. The right upper arm showed weaker autonomous movement after painful stimulus than the other limbs. He showed bilateral equal pupillary circle, light reflex sensitivity, and symmetrical nasolabial folds. Signs of meningeal irritation and the Babinski reflex were absent. Brain computed tomography (CT) showed some attenuation within the left frontal lobe, which was localized to the area of the left middle cerebral artery and was assessed as a 9 on the Alberta Stroke Program Early CT Score. However, CT angiography did not identify culprit blood vessels. Acute cerebral infarction was suspected, so the patient was given anti-platelet, anti-epileptic, and lipid-lowering therapies.

**Figure 1:**
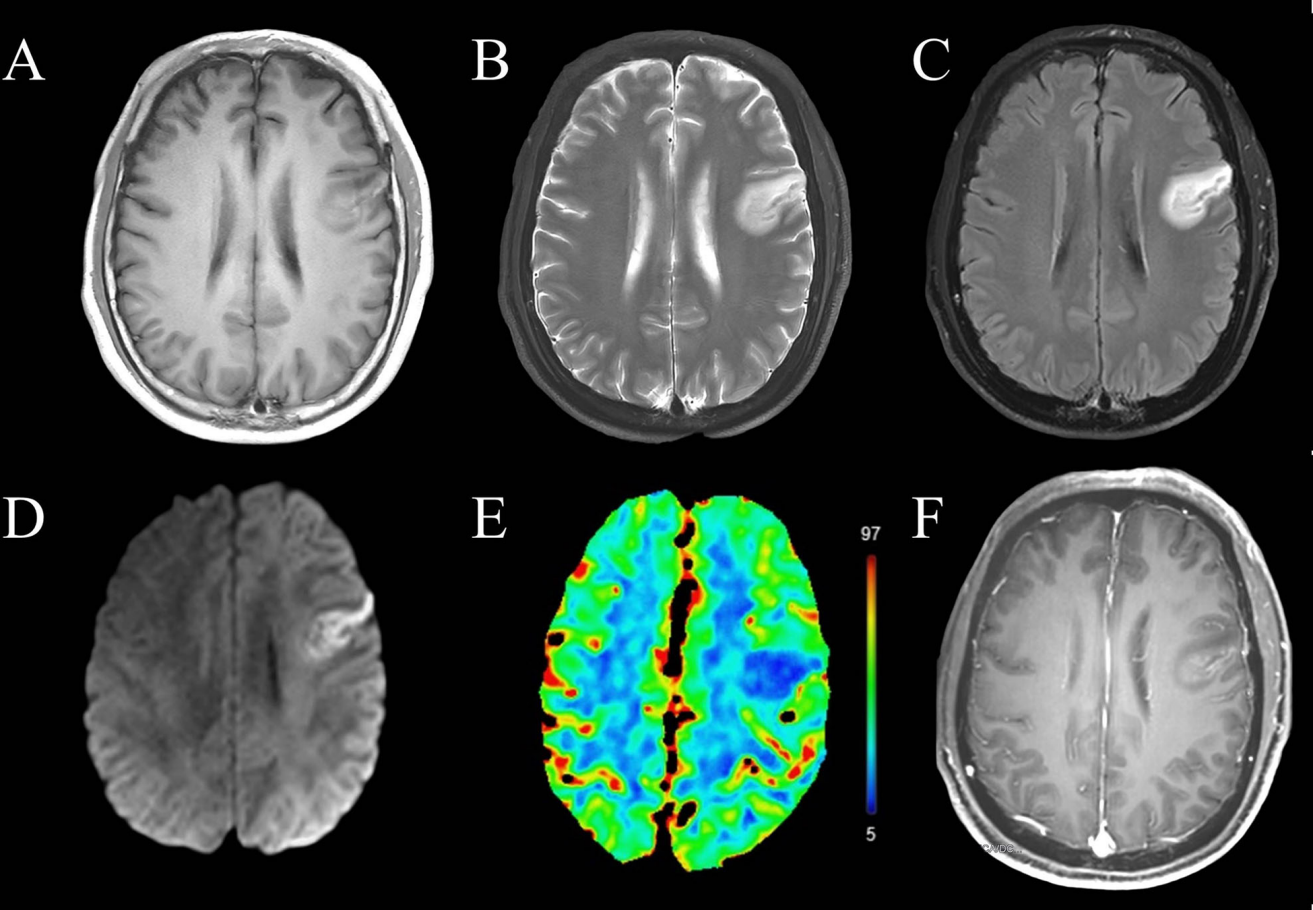
Magnetic resonance imaging of the brain before immunotherapy. The left frontal lobe showed abnormal **(A)** T1 hypointensity, **(B)** T2 hyperintensity, **(C)** hyperintensity on fluid-attenuated inversion recovery imaging, **(D)** restricted diffusion and **(E)** decreased cerebral blood flow. **(F)** This lesion showed gyral enhancement involving the cortex and subcortical white matter.

When symptoms did not improve, the patient was transferred to the neurology department on day 3 after disease onset, and the mini-mental state examination (MMSE) to assess cognitive function showed a score of 20. The patient underwent brain MRI, showing abnormal hypointensity in the left frontal lobe on a T1 sequence, hyperintensity on T2 and fluid-attenuated inversion recovery sequences, restricted diffusion, and decreased cerebral blood flow (**Figure 1**). Contrast-enhanced T1-weighted MRI showed gyral enhancement involving the cortex and subcortical white matter. Chest–abdomen–pelvis CT excluded the possibility of malignancies. Differential diagnoses were considered, including cerebral infarction; encephalitis; mitochondrial encephalomyopathy, lactic acidosis, and stroke-like episodes (MELAS); and cortical venous thrombosis.

Routine laboratory tests, including blood tests, were unremarkable, except for elevated levels of C-reactive protein (80 mg/L; normal range, < 5 mg/L), interleukin-6 (7.31 pg/ml; normal range, 0–7 pg/ml) and d-dimer (1.1 mg/L; normal range, < 0.55 mg/L). Serological tests for infection, malignancy, or autoimmunity were normal, except for a slightly decreased level of IgM (649 mg/L; normal range, 700–2200 mg/L). Analysis of cerebrospinal fluid was normal, and no malignant lymphocytes were detected. No DNA of herpes simplex virus, Epstein–Barr virus, or human cytomegalovirus was detected. A cell-based assay based on HEK293 cells transfected with CASPR2 isoform 1 revealed the existence of anti-CASPR2 antibodies at a titer of 1:10 in blood serum. Antibodies against NMDA receptors, LGI1, AMPA receptors 1 and 2, or GABA_B_ receptors were not detected in serum (Raretest Co., Ltd., Xi’an, China). The patient was diagnosed with anti-CASPR2 antibody-associated autoimmune encephalitis based on the clinical manifestations, epidemiological features, laboratory results and MRI imaging changes.

**Figure 2:**
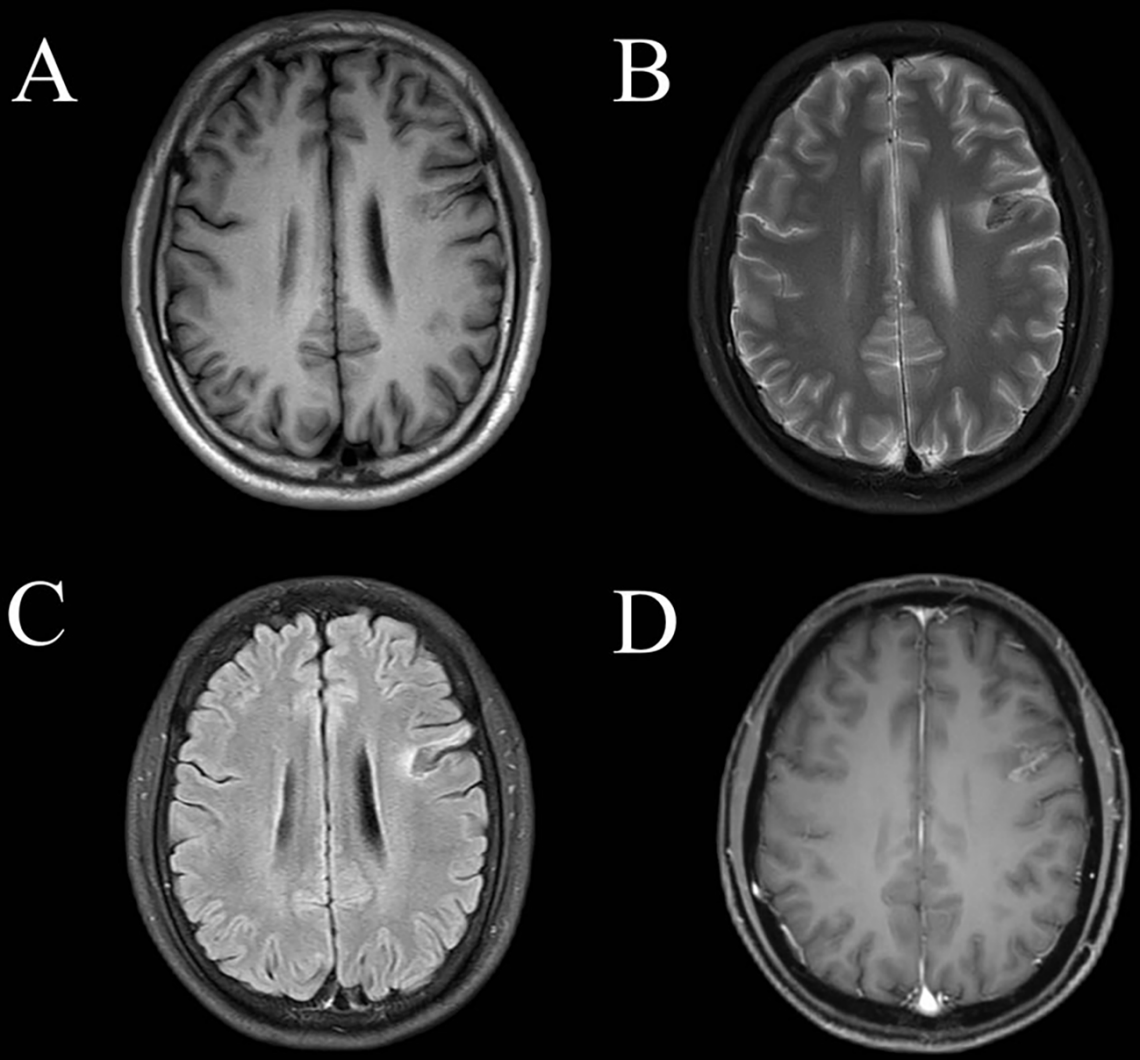
Magnetic resonance imaging of the brain one month after immunotherapy. The left frontal lobe showed abnormal **(A)** T1 hypointensity, high-low mixed sign on T2 imaging **(B)** and on fluid-attenuated inversion recovery imaging **(C)**. **(D)** This lesion showed gyral and patchy enhancement involving the cortex and subcortical white matter.

**Figure 3:**
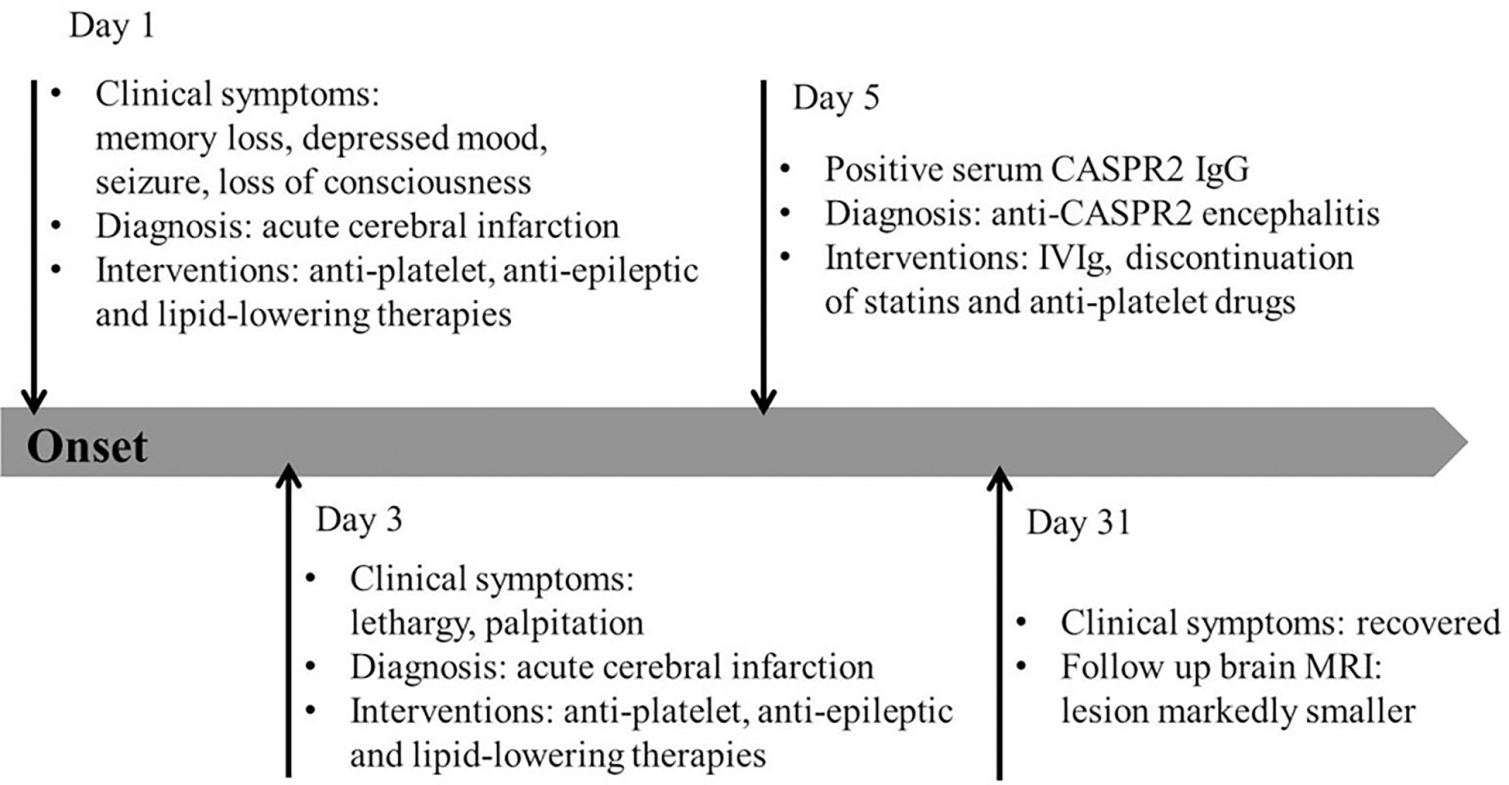
Timeline of the patient’s episodes and interventions. Day 1 refers to the day of admission to our emergency department. CASPR2, contactin-associated protein-like 2; IVIg, intravenous immunoglobulin.

Statins and anti-platelet drugs were discontinued immediately, and the patient was given intravenous immunoglobulin therapy (32.5 g/day for 5 consecutive days). Symptoms began to improve substantially after one week of immunotherapy, except for long-term memory issues. MRI one month after initiation of immunotherapy showed a markedly smaller frontal lobe lesion than before therapy (**Figure 2**). Meanwhile, the MMSE score was 28 one month after initiation of immunotherapy. The timeline of the patient with relevant data regarding the episodes and interventions is presented in **Figure 3**.

## Discussion

Autoimmune encephalitis, while one of the most frequent causes of non-infectious encephalitis, rarely involves antibodies against CASPR2. While detection of such antibodies in serum or cerebrospinal fluid is crucial for definitive diagnosis (10, 11), clinicians may fail to perform such assays or such assays may be unavailable, leading to misdiagnosis with one of several conditions that involve similar symptoms (8). Our case demonstrated the involvement of the frontal lobe in anti-CASPR2 antibody-associated autoimmune encephalitis, with imaging features of gyral enhancement, restricted diffusion, and hypoperfusion, which may be misdiagnosed as the cerebral infarction without thorough analysis.

The present patient was first suspected of having suffered acute cerebral infarction, based on clinical manifestations of memory loss, convulsions, and disturbed consciousness, together with CT and MRI findings. However, CT angiography failed to identify culprit blood vessels, and the patient did not respond to standard treatments for cerebral infarction.

Unfortunately, clinicians initially failed to properly interpret the MRI findings which did not show typical lesions in the medial temporal lobe of anti-CASPR2 encephalitis but mimicked cerebral infarction. Gyral enhancements can be caused by both vascular and inflammatory processes. In cases of infarct, gyral enhancement results from various mechanisms depending on the time course of events (12). Early gyral enhancement can be caused by reversible blood–brain barrier changes when ischemia lasts for only several hours before reperfusion occurs. In subacute stages it appears to be due to reperfusion or vascular proliferation. In encephalitis, gyral enhancement may be due to the breakdown of blood–brain barrier. The primary distinction between vascular and inflammatory causes of this serpentine pattern of enhancement relies on correlation with clinical history and the region of enhancement (12). An abrupt onset of symptoms may suggest a vascular cause, while a more indolent history and nonspecific headache or lethargy may suggest inflammation or infection. Gyral lesions affecting an area around a single artery are often caused by vascular diseases, while inflammatory lesions may affect multiple brain regions. In this case, the MRI manifestations on day 3 after onset mimicked cerebral infarction. Gyral enhancements in the left frontal lobe involving both cortex and subcortical white matter are important signs to remind clinicians of diagnosis of autoimmune encephalitis when the lesion site is not typical for encephalitis.

Anti-CASPR2 antibody-associated autoimmune encephalitis typically occurs around the sixth or seventh decade of life, and most affected individuals are men (6, 13). Studies based on MRI and positron emission tomography have identified the medial temporal lobe as the part of the central nervous system most vulnerable to anti-CASPR2 antibodies (6, 8, 9, 14, 15). However, some cases of abnormalities have been reported in the frontal, parietal, and occipital lobes, brainstem, cerebellar regions, basal ganglia, and the insula (6, 9, 16, 17). Frontal lobe lesions have been detected by positron emission tomography in a 22-year-old woman with systemic lupus erythematosus (18) and a 61-year-old man without systemic disease (19), neither of whom showed abnormal brain MRI. Another case of anti-CASPR2 antibody-associated autoimmune encephalitis showed abnormal T2-weighted hyperintense patchy lesions without restricted diffusion in the subcortical white matters of the frontal lobe, which showed patchy or ring-like enhancement on contrast-enhanced T1-weighted MRI (17). The present patient did not show lesions in the medial temporal lobe by either CT or MRI. Instead, it appeared to be the first reported case of such autoimmune encephalitis affecting the left frontal lobe with gyral enhancement involving the cortex and subcortical white matter. The mechanisms responsible for the different propensity of involved brain regions in the current literature remain unclear. In a previous case of anti-CASPR2 encephalitis with involvement of the brainstem, the authors suggested that the predominant exposure of antigenic epitopes in the brainstem may have facilitated the preferential binding of anti-CASPR2 IgG1 to the target self-antigens and triggered subsequent pathogenic cascades (17). A previous study on anti-GABA_A_ receptor encephalitis reported that the topology of MRI lesions might be associated with the distribution of β3 subunit-containing GABA_A_ receptors and reflected the patients’ disease severity and outcomes (20). This begs the question of whether there is the same mechanism underlying the involvement of the frontal lobe in the present case of anti-CASPR2 encephalitis case. Future studies using animal models or pathological studies in this unique patient subgroup are needed.

This case had some limitations. First, the cell-based assay revealed the existence of anti-CASPR2 antibodies at a titer of 1:10 in serum, which was relatively low. The detection of autoantibodies is crucial for definitive diagnosis of autoimmune encephalitis and differentiation from other mimics. Overinterpretation and misinterpretation of a patient’s presentations and paraclinical study results could affect diagnosis (21). One previous study reported that a CASPR2 antibody serum titer cut-off at ≥ 1:200 had a diagnostic sensitivity of 85% and a specificity of 81% (14). Notably, such a titer of 1:10 has been reported in previous studies in patients with anti-CASPR2 antibody encephalitis (9, 17). Specifically, one study described two patients with this disease with prominent brainstem lesions seen on MRI. These two patients had full or partial neurological recovery after immunotherapy (17). In another study, unfortunately, the authors did not provide detailed information about the patients with titers of 1:10 (9). The clinical manifestations at the onset of disease in the present case included memory loss, depressed mood, seizure, and loss of consciousness, which are consistent with previous cases (6, 13) and met the diagnostic criteria for possible autoimmune encephalitis proposed in the recommendations for a clinical approach to autoimmune encephalitis (10). As mentioned in previous studies, anti-CASPR2 encephalitis occurs mainly in men, which is consistent with the present case (13, 14, 22, 23). After treatment for stroke, symptoms did not improve but began to improve after immunotherapy. Furthermore, MRI one month after initiation of immunotherapy showed a markedly smaller frontal lobe lesion than before therapy. Thus, the clinicians finally diagnosed this patient with anti-CASPR2 antibody-associated autoimmune encephalitis based on the clinical manifestations, epidemiological features, laboratory results, MRI imaging changes, and response to immunotherapy. Long-term follow-up and monitoring are needed in such patients with low titers of antibodies to verify diagnosis. Second, this report is limited by its retrospective nature with incomplete data. Regarding cognitive assessment, only the MMSE scores before and after immunotherapy were available. Unfortunately, no specific formal assessment for memory was conducted. The MMSE is not suited to assess medial temporal dysfunction due to its non-specific nature. Thus, detailed information about the memory of the patient one month after immunotherapy was lacking. Third, considering financial burden for the patient, anti-CASPR2 antibodies and a number of other antibodies were not checked in the cerebrospinal fluid. However, it is fortunate that the clinicians and radiologists corrected the treatment plan from stroke treatment to immunotherapy in time on the basis of thorough analysis of serum, MRI, and clinical symptoms.

## Conclusion

Our case provides the first indication that anti-CASPR2 antibody-associated autoimmune encephalitis can manifest as involvement of the cortex and subcortical white matter in the frontal lobe based on MRI. Gyral enhancement in the left frontal lobe involving cortex and subcortical white matter can aid clinicians in the differential diagnosis of not only cerebral infarction but also autoimmune encephalitis. The present case highlights the need for thorough analysis, including assessment of potential autoimmunity, in patients whose imaging features mimic ischemic infarction.

## Data availability statement

The original contributions presented in the study are included in the article/supplementary material. Further inquiries can be directed to the corresponding author.

## Ethics statement

The studies involving human participants were reviewed and approved by Ethics Committee of West China Hospital of Sichuan University. The patients/participants provided their written informed consent to participate in this study.

## Author contributions

ZC and JT collected data and drafted the manuscript. All authors contributed to the article and approved the submitted version.
